# Huntington’s Disease Community Perspectives on Desired Characteristics of Disease Modifying Therapies

**DOI:** 10.5334/tohm.584

**Published:** 2021-01-20

**Authors:** Michele C. Gornick, Kerry A. Ryan, Praveen Dayalu, Noelle E. Carlozzi, Roger L. Albin, Darin B. Zahuranec

**Affiliations:** 1 Center for Bioethics and Social Sciences in Medicine, Michigan Medicine, US; 2 Department of Neurology, Michigan Medicine, US; 3 Department of Physical Medicine and Rehabilitation, Michigan Medicine, US; 4 Geriatrics Research, Education, and Clinical Center, VAAAHS, US

**Keywords:** outcome measures, benefits, harms, qualitative, Huntington disease

## Abstract

**Background:**

Promising disease modifying therapies for Huntington’s disease are now entering pivotal trials, raising questions of what patients and families consider successful outcomes. Consistent with an ongoing movement to incorporate patient preferences into the development of new therapies, we conducted a pilot study to assess Huntington’s disease community views on emerging DMTs to assist in planning large-scale studies of patient preferences.

**Methods:**

Semi-structured interviews were conducted with members of the Huntington’s community (manifest disease, at-risk, and family/caregivers). Participants were asked which symptoms they believed should be targeted with novel treatments, as well as potential benefits and tradeoffs of delaying symptom onset versus prolonging late-stage disease.

**Results:**

Participants (N = 14) emphasized the need for treatments improving cognitive and/or behavioral symptoms. Many wanted treatments that delayed symptom onset up to 5–10 years, though some considered shorter delays acceptable due to potential value in advancing research to help future generations. Concern regarding potential for prolonging later-stage disease was variable, with some participants uncertain if they would want a treatment that delayed onset but prolonged later-stage disease. Others stated that any delay in onset would be desirable, regardless of potential prolongation of later stage disease.

**Discussion:**

This study demonstrates a breadth of opinions among the Huntington’s disease community surrounding both the benefits and complex tradeoffs that might occur with disease modifying treatments. These preliminary findings will inform future large-scale studies of attitudes toward disease modifying treatments, which may ultimately guide the design and outcome measure selection for clinical trials.

**Highlights:**

In-depth interviews with the Huntington’s disease community were used to explore patient and family preferences regarding potential disease modifying therapies. Many wanted symptom delay of 5–10 years, though some considered shorter delays acceptable for altruistic reasons. Opinions on trade-offs varied, suggesting larger preference studies are needed to inform trial design.

## INTRODUCTION

Current clinical management of Huntington Disease (HD) focuses on the treatment of motor and psychiatric symptoms with limited efficacy [1,2]. Translational research has advanced to the point that several targeted disease-modifying treatments (DMTs) are currently under development. DMTs based on suppression of mutant *huntingtin* gene expression showed promise in pre-clinical and early phase studies with pivotal trials starting for anti-sense oligonucleotide therapy [3,4,5].

As potential DMTs were being developed, the United States Food and Drug Administration (FDA) recognized the importance of engaging the HD community to understand patient and family perspectives on disease features and treatments. A 2016 FDA report found that HD community members felt that that current treatments did not adequately manage the cognitive or behavioral symptoms that are characteristic of the disease. They also emphasized the need to pursue treatments that delayed (or cured) HD [6,7].

The FDA report is an important first step in understanding patient and family perspectives, but several important questions remain unanswered. In particular, this report did not explicitly address potential trade-offs between expected beneficial and potentially harmful effects of DMTs. In simulation studies, we showed that treatments that delay onset of HD may result also in patients surviving longer during the symptomatic phase with manifest HD [8]. How the HD community views such trade-offs is critical for future DMT trial design for several reasons, including informing selection of appropriate outcome measures, as well as anticipating potential challenges in recruitment. With initial pivotal trials already underway, and the growing likelihood of complex treatment decisions in HD in the near future, now is the appropriate time to engage the HD community to understand their priorities about the criteria for a successful intervention [9] and how they view the possibility that some promising treatments might produce lasting negative effects.

Addressing these questions is complicated by the multifaceted structure of the HD community, as individuals with manifest HD, those at risk for HD (both mutant allele carriers and non-mutant allele carriers), and family members might have different opinions. Furthermore, those with manifest HD may experience disease burden than impacts their ability to make informed choices. We used semi-structured, in-depth interviews to conduct a preliminary assessment of these varying perspectives to assist in the planning of future larger scale studies of HD community preferences and attitudes toward DMTs.

## METHODS

### PARTICIPANT RECRUITMENT

HD patients, at-risk individuals, family members, and caregivers were recruited from a University Movement Disorders clinic either in person, via posted flyers in clinic, or via informational letters. Participants had to be the age of 18 or older and fall into one of these categories: HD patients (diagnosed), at-risk (e.g. affected first degree family member), or family members/caregivers of an individual with HD. Purposive sampling was used to obtain approximately equal representation from these three groups. HD subjects were screened using the Orientation Log-HD to assess their cognitive capacity to consent for participation (score >= 25 eligible) [10,11]. Although there was no formal assessment of HD staging, this cognitive screening requirement meant that all participants with manifest HD were effectively in early stages of disease (scores for participants with manifest HD ranged from 28–30 out of 30). This study was reviewed and approved by the local Institutional Review Board and participants provided informed consent prior to the participation in any study related activities.

### DATA COLLECTION

We used a mental models approach to our semi-structured interviews [12]. This approach seeks to understand the mental frameworks that participants use to understand the topic of the interview (in this case, HD symptoms and DMT), and then use interviewees’ own mental frameworks to discuss the relevant issues. The goal was to allow each participant to describe their experience with HD on their own terms, therefore making the interview more meaningful and engaging. The initial portion of the interview was an open-ended account of each individual’s personal experience with HD. Based on the individual’s self-described experience with HD, subsequent questions about disease progression, symptoms, DMTs, and tradeoffs were tailored to the individual’s mental framework of HD. Participants were asked, for example, “What would be a good [bad] change in HD for you?” Participants were also asked about “good/bad” HD related symptoms (i.e., behavioral, cognitive, motor) changes based on each individual’s experience with the disease. Based on participant’s personal experiences and responses to earlier questions, the interviewer would probe with: “What if this treatment [insert the bad change in HD] but not [insert good change in HD]. To elicit views specifically on tradeoffs that may occur with these future disease-modifying therapies, interviewers probed about desired delay of onset and thoughts about prolonging later stages of HD. Interviews were performed by trained interviewers (MCG, KAR) using a semi-structured interview guide (see Supplementary Appendix for interview guide). Interviews lasted approximately 1 hour and were conducted in a private conference room, via telephone, or in participant homes. All interviews were audio-recorded, transcribed verbatim, and de-identified for analysis.

### DATA ANALYSIS

De-identified transcripts were analyzed in MAXQDA (VERBI Software, 2017), a qualitative data analysis software program that assists with searching text, coding thematic domains, and organizing data. The qualitative analysis team (KAR, MCG, DBZ) independently read the first few transcripts to generate preliminary thematic domains for coding; this group met regularly to review coding consistency and assess new codes and emerging topics. Differences in coding were resolved by consensus. New codes were applied to all transcripts iteratively. Codes were further aggregated into a number of broad thematic domains of interest and representative quotations were identified. Direct quantitative comparisons across interview or participant characteristics are not provided for several reasons. The goals of this work are exploratory, and the sample size precludes definitive quantitative comparisons. Furthermore, due to our efforts to tailor questions to the participant experience, there were minor variations in how certain topics were assessed. Participant identification (ID) and category within the HD community are provided after each quotation to give a general sense of the breadth of responses in the sample. Descriptive statistics were used to summarize the participant characteristics.

### RESULTS

Out of 17 eligible individuals who initially agreed to be interviewed, 14 (83%) completed the interview. Participant characteristics are shown in the ***Table***.

**Table:** Participant Characteristics (n = 14). SD = Standard deviation; HD = Huntington’s disease.


CHARACTERISTIC	N (%) OR MEAN (STANDARD DEVIATION)

**Gender**	

Female	8 (57%)

Male	6 (43%)

**Age, Mean (SD)**	48.6 (15.9)

**Ethnicity**	

Non-Hispanic White	14 (100%)

**Education**	

More than Bachelor’s degree	2 (14%)

Bachelor’s degree	4 (29%)

Less than Bachelor’s degree	8 (57%)

**HD Status (All that Apply)**^1^	

At risk	3 (21%)

Caregiver	4 (29%)

Gene-negative	1 (7%)

Gene-positive (e.g. pre-manifest)	3 (21%)

Manifest HD	4 (29%)


^1^ Numbers sum to 15 as 1 individual was both a caregiver and at-risk.

The data from the interviews are presented as responses to three primary questions relevant to future DMTs: (1) What are the most important symptoms to target?; (2) How much of a delay in disease onset is enough?; and (3) What if delay of onset also leads to prolonging the later stages of the disease?

### WHAT ARE THE MOST IMPORTANT SYMPTOMS TO TARGET?

Participants discussed their views about the impact of motor, cognitive, and behavioral symptoms of HD on their lives or the lives of their loved ones. They also discussed which types of symptoms were most critical for a potential treatment. Participant views tended to center around two primary themes: (1) cognitive and behavioral symptoms were most important, or (2) *all* of the symptoms were too interconnected to target just one.

Some participants indicated that improving cognitive and/or behavioral symptoms would be more important for them than motor symptoms.

… if I had to pick between the mental…you know, being alert and understanding what’s going on and…that part of things as opposed to being in a wheelchair, I would want my brain intact so to speak. (ID 2, At-risk)

I think that I myself would rather deal with maybe the movements and have the clear memory, the better mood… (ID 11, Premanifest mutant allele carrier)

Other participants indicated that all of the symptoms were interconnected and could not be ranked in terms of importance when developing a treatment.

I feel like those two things [movement and memory] are so interconnected. I don’t really feel like that would be good because if you lose your ability to remember what you’re doing or think about what you’re doing, then if you can move your body but you can’t get your mind to do anything that doesn’t seem like it would be any sort of progress. […] I would hope to be something that would be comprehensive because all of the symptoms are so connected. (ID 10, Premanifest mutant allele carrier)

It’d be a coin toss between the physical and the cognitive changes, but, yeah. Physically, if you can move around well enough, you can take care of yourself for the most part. I don’t want to be sitting on the…you know, totally like in a wheelchair and be mentally okay. There’s no good… No good combination, I guess. (ID 16, Premanifest mutant allele carrier)

Though participants also discussed the importance of motor symptoms, only one individual seemed to consistently indicate that he was most concerned about motor or physical symptoms of HD.

*It’s tough, but the physical thing is really tough. That’s when you gotta start, you know, doing everything for them and to me, that’s the worst part*.

Interviewer: In your opinion, if a treatment could change and improve only one thing, what would be the most important thing to you or to your family?

Well, that would be the physical. (ID 14, Caregiver)

### HOW MUCH OF A DELAY IN DISEASE ONSET IS ENOUGH?

Participants were asked about what amount of delay in onset of disease would be necessary for them to consider a new treatment to be successful. Participants provided a variety of responses to this question. However, most participants discussed delay of onset in in terms of *years* rather than *months*.

Some participants felt that a treatment would need to substantially delay symptoms (five to ten or more years) for them to consider a treatment be beneficial.

Probably like a minimum of 10 years. […] I mean, hopefully longer, but…

Interviewer: What if the treatment was 5 years more?

I feel like anything is useful compared to nothing. It’s like again, it’s hard for me to just be like that would not be helpful at all because it’s still guaranteeing like 5 years with no symptoms, so that’s still a good thing. I mean, I’d prefer if it was like a 50-year delay… (ID 10, Premanifest mutant allele carrier)

Interviewer: …how much of a delay would it need to be for you…to consider a treatment to be successful?

*Ten years*.

Interviewer: Okay, what about five years?

*Five years would be great too, yes*.

Interviewer: Two?

No… Yeah, five to ten. (ID 12, Caregiver)

…if it adds 10 years, I mean right now I’d be 65 to 75. A lot of people don’t make it to 75, so I mean it’s really not a bad deal… (ID 7, Manifest HD)

One participant seemed to indicate that a longer delay of onset would be needed to offset concerns about discrimination around predictive test results.

… Probably 5 to 10 years, probably 10 years. I don’t know. I’m not against getting tested. I get scared to get tested sometimes because I’m going to school and I get scared of discrimination. (ID 6, At-risk caregiver)

Others felt that shorter-term delays (of less than 5 years) would still be of value.

The delay, the…progression of it. I understand that would make it longer, but if we had instead of 3 months of you know, of balance, we’d have a year and a half. That’s huge. That makes a you know, more quality of life. (ID 2, At risk)

Some participants specifically indicated that a shorter delay would be acceptable as it may have value in advancing research to help future generations.

I mean, in my opinion, anything. I think like I said before, those small strides. Even if something is a potential now that might improve just a little bit, I think that a small improvement now might give the basis of providing a larger improvement later. So, I think that even something that only offers something small to me might in twenty years offer something large to my children. (ID 11, Premanifest mutant allele carrier)

*If there was a delay for it, I would be very likely to look into that and be willing to try it. I mean, with…the whole thought is if it helped with research and we were getting closer to being able to make it so that Huntington’s almost never showed any symptoms, that would make a better life for my kids or my nieces or nephews or…or whatever. That would be very important*.

Interviewer: What if the treatment […] only delayed a year or 2 years? Would you still…?

Consider it? Yeah. (ID 2, At risk)

One participant felt that a shorter delay of onset was acceptable as long as treatment regimen was not too difficult.

I think that, if you had a good quality of 5 years, that’s giving you 5 more years. Interviewer: What about 2 or something like that? […] I don’t know if there’s a deadline. I mean I guess it depends on what’s involved with the treatment and what does the patient have to go through […] If it’s something that’s difficult for the patient to go through and it’s going to give them 1 or 2 more years, I don’t know. (ID 17, Non-mutant allele carrier)

One participant felt that even if a treatment provided a longer delay (of 10 years), its value depended on the health-related quality of life of the patient with HD.

I guess it just depends at what level you get you live, like if you can spend an extra 10 years in a nursing home, no thanks. If you can spend another 10 years living a normal life, then that sounds good. […] I think it would just depend on whether or not you can still do things well. If you can do stuff and you’re basically normal with medication, then that’s…I think that’s an improvement but if you still have to be in a stage that you can’t work or you can’t drive or anything like that, then I don’t feel like that would be as productive or as successful, but if you were still able to maintain a normal life because you take a lot of medication, I mean that’s better than nothing. (ID 10, Premanifest mutant allele carrier)

### WHAT IF DELAY OF ONSET LEADS TO PROLONGING THE LATER STAGES OF HD?

Participants also discussed the potential tradeoff between delaying onset of HD symptoms and prolonging the later stages of the disease.

One participant felt that the value of a treatment that delayed onset really depended on whether or not it led to prolonging of the late stage of HD. To him, any delay was acceptable as long as it did not prolong late-stage disease.

Well, if it extended the end, that’s you know…that’s a big price to pay, but I mean, obviously, if it doesn’t extend the end stage, any sort of delay would be a great delay obviously. But, yeah, if I had to pay for it on the other end, I don’t know [Later] Interviewer: … what if there was no extend in the end? What if it just delayed things now?

Yeah, absolutely. I’d be 100 percent on board with doing whatever delays it… Interviewer: Even if it was two years or…?

Yeah. […]

Interviewer: Yeah, what about…yeah, six months?

Anything. Yup, if there’s no extension at the end. I just don’t want that end stage to be long for my wife and I don’t want it to be long for me. (ID 16, Premanifest mutant allele carrier)

Another participant was willing to accept potential negative consequences at later stages of HD, if it helped delay symptoms now, as long as the delay was terms of years, not months.

… if it only helped for like 90 days, well then, it’s not worth it. […] If it was going to delay it for a couple years or 5 years then make it come on faster, I guess it’d be worth it. (ID 9, Manifest HD)

Other participants were less concerned about a treatment prolonging the later stages of HD if it delayed onset of the disease, as they could always refuse life-sustaining treatment (such as a feeding tube) later.

… as much time as I can have being healthy, I’d want to be healthy. I mean, I can always choose early on if I don’t…if I want a feeding tube or not and…I mean, I won’t live a long time without one. (ID 6, At risk caregiver)

If it helped out now, then yeah, because then…well if it was that bad where I had a feeding tube, I’d have to say that I’d just let go. (ID 5, Manifest HD)

## DISCUSSION

In this exploratory pilot study of the HD community, we report heterogeneous opinions about desired characteristics of DMTs. While these findings are preliminary because of the small sample size, these results demonstrate the importance of engaging the HD community at large on the criteria for successful DMTs. Key findings, which warrant confirmation in larger samples, included the relative importance of ameliorating cognitive or behavioral symptoms, and several participants’ desire for new treatments to delay the onset of manifest HD by years rather than months. This work is consistent with a larger movement to incorporate patient preferences into the drug development process [9], and our findings can help to inform the design of future larger scale studies of treatment preferences.

It is worth noting the distinction between studies of patient preference information and other assessments based on patient input, such as patient-reported outcomes [13]. A patient reported outcome measure is designed to allow the patient to directly report on their health status or symptoms. In contrast, patient preferences studies assess the relative desirability of particular health interventions or outcomes and provide information on what the patient wants. Thus, these multiple types of patient input can provide complementary information.

Study participants tended to emphasize the importance of targeting cognitive or behavioral symptoms of HD. While motor symptoms were also important, they were generally considered interconnected with non-motor problems. The priority that respondents placed on cognitive or behavioral symptoms is consistent with the recent FDA report on attitudes towards HD treatments [6,7]. While this finding is not novel, it was necessary for us to include this topic to understand respondents’ mental models of HD, and the consistency with the FDA report is reassuring.

Many individuals in this study indicated that they are seeking treatments that delay HD onset by 5–10 years, suggesting enthusiasm for treatments with large and robust treatment effects. Other respondents felt that shorter delays in HD onset would be acceptable. Some participants specifically commented that even small improvements now might lead to subsequent therapies that would be more effective for future generations. This consideration of benefit to family members is consistent with previous work in dominantly inherited neurodegenerative conditions that identified altruism as a motivation for specialty clinic attendance [14]. Further exploration of how this altruistic attitude may impact willingness to participate in clinical trials is warranted as many members of HD families are likely acutely aware of its multi-generational impact.

It is worth noting that our line of questioning about desired delay in disease onset is primarily relevant to individuals with pre-manifest disease. In contrast, present DMT trials enroll participants with manifest HD, which is necessary given the problem of exposing healthy individuals to interventions of uncertain benefit and unknown risks. As such, these trials use clinical endpoints such as the Total Functional Capacity (TFC) score or the Unified Huntington Disease Rating Scale (UHDRS) [15]. These measures lack sensitivity and clinical utility in the premanifest stage of HD making it impossible to extrapolate trial outcomes based on these clinical endpoints to estimate the degree of benefit in at-risk/premanifest populations.

Intuitively, if a DMT had a robust effect in clinical rating scales in manifest HD, we might expect it to have a large effect in terms of delaying onset (e.g. years rather than months) in pre-manifest disease. It is not certain, however, that the drivers of neurodegeneration in manifest and pre-manifest HD are identical. The former might involve secondary neurodegenerative cascades, such that a modest effect in manifest HD might correspond to a disproportionately larger effect in pre-manifest populations. Similar considerations apply to surrogate endpoints, such as MRI morphometry, that might be used in DMT trials in at-risk/pre-manifest populations. Notwithstanding these concerns, it is important to confirm a broader desire within the HD community to delay manifest disease onset by years, as this would point to pursuing smaller trials predicated on larger effect sizes, which might speed up DMT development. These considerations and our results underscore the need to better understand the biology of HD in pre-manifest populations, and to develop clinically meaningful methods to assess HD progression in at-risk/pre-manifest populations.

Regarding the possibility that DMTs might prolong later stage disease, responses varied widely. Some respondents expressed great concern about such tradeoffs; others were less concerned. We did not have a large enough sample to understand reasons for these differences in level of concern. Some respondents clearly considered this issue in a sophisticated manner, as they considered the idea that a long enough delay in manifest disease onset could result in death from a later, competing cause of mortality. Others suggested that they may consider forgoing life-sustaining treatments, such as artificial nutrition, if they were facing prolonged late-stage disease. Further understanding of how patients may react to potential prolongation of poor quality of life will be critical, particularly with expanded access to medical assistance in dying in many parts of the world [16]. Due in part to time constraints in the interview, discussions of this topic tended to focus on the marked impairments of late stage disease, rather than on the possible consequences of prolonging HD in earlier stages, a period when behavioral features are often prominent. Future work will need to parse out HD community concerns about specific tradeoffs in different phases of HD with the use of DMTs.

The HD community is diverse, including individuals with manifest disease, individuals with known mutant allele carrier status, at-risk individuals ignorant of mutant allele carrier status, and family members with and without caregiving roles. Even though our exploratory study results are consistent with heterogeneous perspectives in the HD community, we were not able to reach meaningful conclusions about similarities or differences between important constituencies of the HD community. Even within a group, perspectives may vary widely. HD patients, for example, vary considerably in cognitive impairments and insight into the severity of their disease. In addition, the mental models approach for interviews, while useful for exploring participant experiences and perspectives, resulted in non-uniform format of some of the questions posed. Future work with a larger heterogeneous sample and a more structured set of questions is needed to better understand the full spectrum of treatment preferences in the HD community and across important groups before applying any of these findings to clinical care or trial design.

This study has several limitations, including the single-center recruitment, lack of formal assessment of baseline knowledge of HD progression, and the small sample size. While the sample size is the primary limitation, sample sizes in qualitative research are commonly smaller than in quantitative studies [17] including multiple prior studies in HD [14,18,19]. Based on the sample size, any findings in this preliminary study may not be generalizable and would require confirmation in larger samples. This study can directly inform these future studies in several ways. First, members of the HD community were interested and able to engage on these issues of complex tradeoffs, suggesting that future study in this area is both feasible and warranted. Second, this study has provided perspectives on several important domains (altruism, desire for highly effective treatments, concerns about genetic discrimination, and refusing artificial nutrition at the end of life) that seem worthy of exploring in future studies of attitudes toward DMTs. This future work should also include investigation of other important domains that we were not able to address in this study such as side effects, mode of delivery, duration of treatment, and costs. Additionally, future studies may want to focus more on those with early manifest disease most likely to be eligible for DMTs. We are currently planning additional qualitative research with a broader coalition of stakeholders to better understand the critical domains of DMTs tradeoffs to study. This qualitative work can then facilitate development of quantitative measures suitable for a more widespread assessment of HD community preferences toward DMTs that can inform future clinical trials and facilitate real-world implementation of DMTs.

## ADDITIONAL FILE

The additional file for this article can be found as follows:

10.5334/tohm.584.s1 Supplementary appendix. Overview of interview guide.
