# Supplementary material for: Huntington’s Disease Community Perspectives on Desired Characteristics of Disease Modifying Therapies
Source: Tremor Other Hyperkinet Mov (N Y). 2021 Jan 20;11:3. doi: 10.5334/tohm.584 (PMC7845464; doi:10.5334/tohm.584)
Supplement: Supplementary appendix. — Overview of interview guide. [file tohm-11-1-584-s1.pdf]

Supplementary appendix: Overview of interview guide

The interview guide used in this manuscript is provided on the subsequent pages. Tailoring of the interview to participant type is indicated in the brackets. Note that this manuscript is focused primarily on research question 2, though research question 1 is also provided as a reference.

> *Research Question 1: How do HD participants understand and describe symptom progression and stages of disease?*
>
> *Research Question 2: How do HD participants view emerging treatments for HD, particularly considering tradeoffs that may occur with these novel treatments?*

Research Question 1

1. [All] Please tell me the story of your experience with HD.

   Probes (or make sure story includes responses to all):
   - Who in your family has/had HD?
   - Tell me about your experiences with this/these family member(s).
   - At what age did you/your family member(s) start showing HD symptoms?
   - Do you have experiences with others outside your family with HD? Tell me about that.

2. [If at risk for HD or has HD] When did you find out that you were at risk for HD? Tell me about your experience with finding out. (How did it make you feel?) *[Alternate: [If family member] When did you find out that your family member was at risk for HD? Tell me about your experience with finding out. (How did it make you feel?)]*

Now I am going to ask you about the impact of HD symptoms on persons with HD, their family and caregivers.

3. Each person and each family has a unique experience with HD. Are there any [clues/characteristics] that point toward HD or are associated with HD changes [in your family]?

4. What do you think happens to someone who has HD over time, from when it first starts until later over the course of their life?

   Alternative: Please describe what you know about how HD changes over time?

   Probes (if not mentioned):
   - How do motor function/movement symptoms change over time?
   - How do thinking and memory issues change over time?
   - How do social, emotional, or behavioral issues change over time?
   - How does the impact of HD on daily life change over time?

5. [If at risk for HD or has HD] What do you do now that is important to you, that you want to continue doing?) *[Alternate: [If family member] What does your family member do now that is important to you that you want them to continue to be able to do?*

   Probes (if examples are needed):
   - Like doing things for yourself?
   - Like doing things with your family or friends?
   - Like doing things for work or for fun?

6. [If at risk for HD or has HD] What do you do now that you are worried about losing your ability to do? *[Alternate: [If family member] What does your family member do now that you are worried about them losing the ability to do?*

7. What are you worried will happen [to you/to your family member] in the future?

Research Question 2

As you may know, researchers are trying to develop treatments that target the genes responsible for HD. It is unlikely that these treatments would completely cure HD.
We also don't know how these treatments will change HD.

For example, a treatment may reduce or delay some type of symptoms, but not other symptoms. Or delay the start of symptoms but then make the overall course of HD longer. It's also possible that a treatment may only reduce symptoms a little bit or delay symptoms only for a short while. We want to get your thoughts on these issues.

8. What would be a good change in HD for you?

9. What would be a bad change in HD for you?

   Probes:
   - Okay, tell me more about that.
   - How long of a delay, how much time?
   - What would the treatment need to do for motor function/movement symptoms, if anything?
   - What would the treatment need to do for thinking and memory issues, if anything?
   - What would the treatment need to do for social, emotional or behavioral issues, if anything?

10. What if this treatment [insert the bad change in HD] but not [insert good change in HD]?

11. What if this treatment could delay the start of HD by 5 years, but this meant that you would live 5 years longer with the symptoms of HD?

12. What if this treatment only affected some of the symptoms of HD but did not improve other symptoms?
    - If they ask what symptoms, probe on what they would think might me important to them.

People at risk for HD have the option to get a predictive genetic test to find out whether they carry the HD mutation. Some people at risk for HD decide to get this genetic test and some people at risk for HD decide <u>not</u> to get this genetic test.

13. How do you feel about people at risk for HD being required to take the predictive genetic test before they could receive these new treatments for HD?

14. [If at risk for HD, but not tested] How effective would the treatment have to be in delaying or reducing symptoms, for you to consider predictive genetic testing for yourself?

Post-interview Demographic Questions

Are you Hispanic or Latino(a)?

☐Yes
☐No

What is your race? (Please select all that apply)

☐ American Indian or Alaska Native
☐ Asian or Asian American
☐ Black or African American
☐ Native Hawaiian or Other Pacific Islander
☐ White or European American

What is the highest level of school you have completed?

❍ Less than BA
❍ BA
❍ More than BA

Sometimes after we review your answers, we may want to ask you a few more questions to follow up on what you have said. If that happens, would you mind if we contact you to ask a few brief follow up questions? ☐ Yes ☐ No

Would you be interested in being contacted for any future studies? ☐ Yes ☐ No

Would you like to hear about any published results from this study? ☐ Yes ☐ No

[If yes to above] What would be the best way to contact you? [Get telephone/email address]

I have a list of counseling resources we offer to everyone we interview because we know what you are going through can be very difficult.

- [If on phone] Do you have a pen to write a number down?
- [If in person] Hand the sheet to the person.

If you are interested, talking to one of these counselors may be helpful with everything you are going through. If you don't want to talk to a counselor, you could also talk to your primary care doctor, or [if applicable] a religious leader in your community.

Thank you so much for taking the time to talk with me today.
